# Relevance of sonographic parameters for inflammatory bowel disease in children

**DOI:** 10.1007/s40477-023-00800-9

**Published:** 2023-07-04

**Authors:** Alessandra Dell’Era, Rosanna Cannatelli, Francesca Ferretti, Cristina Manzotti, Dario Dilillo, Gianvincenzo Zuccotti, Fabio Meneghin, Sandro Ardizzone, Giovanni Maconi

**Affiliations:** 1 Present Address: Gastroenterology Unit, Department of Biomedical and Clinical Sciences, University of Milan, Via Giovanni Battista Grassi, 74, 20157 Milan, Italy; 2 Department of Pediatrics, Vittore Buzzi Children’s Hospital, University of Milan, Via Lodovico Castelvetro, 32, 20154 Milan, Italy

**Keywords:** Inflammatory bowel diseases, Ultrasound, Fecal calprotectin, Intestinal ultrasound, Children, Bowel wall thickness

## Abstract

**Purpose:**

Intestinal ultrasound (IUS) is widely used as the first exam in patients with suspected inflammatory bowel disease (IBD). This study investigated the accuracy of several IUS parameters, including increased bowel wall thickening (BWT), in detecting IBD in a paediatric population.

**Methods:**

The study included an unselected series of 113 patients aged 2–18 years (mean age 10.8 years, 65 male), referred for recurrent abdominal pain or altered bowel habits, without known organic diseases, to perform an IUS as first investigation of a diagnostic workup. Patients with full systematic IUS examination, clinical and biochemical exams, and ileocolonoscopy or an uneventful follow-up at least one year follow up were eligible.

**Results:**

23 IBD patients (20.4%; 8 ulcerative colitis, 12 Crohn’s disease and 3 indeterminate colitis) were diagnosed. We found that increased BWT > 3 mm (OR 5.4), altered IUS bowel pattern (IUS-BP, OR 9.8) and mesenteric hypertrophy (MH, OR 5.2) accurately identified IBD at the multivariate analysis. IUS-BP, MH and BWT > 3 mm had a sensitivity of 78.3%, 65.2% and 69.6% and a specificity of 93.3%, 92.2% and 96.7%, respectively. The combination of these three alterations increased the specificity up to 100%, whilst decreased sensitivity to 56.5%.

**Conclusion:**

Among several US parameters suggestive of IBD, the increased BWT, MH and altered echopattern are independent predictors of IBD. The ultrasonographic diagnosis of IBD could be more accurate if relied on combination of different sonographic parameters, than on the sole BWT evaluation.

## Introduction

Inflammatory bowel diseases (IBD), including Crohn’s disease (CD), ulcerative colitis (UC) and IBD-unclassified (IBD-U), are characterized by chronic inflammation of the gastrointestinal (GI) tract. Their incidence and prevalence have dramatically increased worldwide over the past 50 years [1]. In up to 25% of cases the onset of IBD is during childhood [2] and disease phenotype is more aggressive and extensive than in adult-onset patients [3, 4]. Furthermore, IBD in children may present with non-specific GI symptoms, frequently overlapping with functional bowel disorders which make diagnosis more challenging and results in diagnostic delays. Indeed, a quarter of children is diagnosed with a delay more than 1 year [5], with an increased risk of IBD progression, complications and need of surgery [3, 4].

Therefore, prompt investigations, are recommended in all children with strong suspicion of IBD, in particular ileocolonoscopy [6]. However, due to its invasiveness (it usually requires hospitalization and general anaesthesia) and high costs, the use of ileocolonoscopy in paediatric clinical practice is limited. Among non-invasive and widely available investigations, European Society for Paediatric Gastroenterology Hepatology and Nutrition (ESPGHAN) guidelines highlighted the use of faecal inflammatory markers and intestinal ultrasonography (IUS) as preliminary or first tests to identify children needing for more invasive investigations.

The accuracy of IUS in the detection of IBD has been well established by systematic reviews, meta-analysis and international guidelines, but data on its accuracy rely only on one parameter such as the presence of an abnormal bowel wall thickening (BWT), namely a BWT more than 3 or 4 mm [7, 8]. However, it is well known that, besides BTW, IUS can detect many other findings suggestive of IBD, such as lymph node enlargement and mesenteric hypertrophy (MH). The assessment of these IUS findings can corroborate or exclude the diagnosis of IBD.

To date, no data are available on the usefulness of combined IUS finding to discriminate children at high risk for IBD. Moreover, the relevance of increased BWT and the additional value of other IUS parameters in the diagnosis of IBD have not been investigated so far. This study aims at investigating the diagnostic accuracy of several US parameters in detecting IBDs in a paediatric population.

## Materials and methods

### Patients

This is a retrospective study including a consecutive series of children aged 2–18 years, referred to our paediatric gastroenterology clinic from 2007 to 2013 for recurrent abdominal pain and/or altered bowel habits (diarrhoea or/and constipation) for whom the decision to proceed with endoscopy was not already definite.

Children were eligible for the study if they had undergone IUS because of altered laboratory tests including faecal calprotectin (FC) and biochemical inflammatory indexes (BII), in particular C-reactive protein (CRP), within 1 week following the initial outpatient visit, as part of a scheduled diagnostic work-up used in our paediatric gastroenterology unit since 2007. Indeed, since 2007 a work-up algorithm based on IUS, BII and FC is used for every child who present symptoms like recurrent abdominal pain and/or altered bowel habits (diarrhoea and/or constipation) and/or history of weight loss with fever.

Exclusion criteria were: (i) known organic diseases (e.g. previously known IBD or celiac disease), (ii) highly suspected IBD (e.g. perianal disease or rectal bleeding), which would have required a digestive endoscopy anyway, or, (iii) a recent (within 6 months) instrumental diagnostic investigations (e.g. upper or low\er endoscopy).

Reference gold standard for IBD diagnosis was the confirmation by endoscopic and/or radiologic investigations performed within 1 year from initial non-invasive diagnostic work-up, according to ESPGHAN revised Porto criteria [6]. Conversely, negative investigations and/or a non-IBD diagnosis after appropriate diagnostic work-up (e.g., celiac disease, food allergy, infections) and a further minimum of 1-year clinical follow-up were considered reference criteria to exclude an IBD.

Being a retrospective study, a formal approval by our local Ethical Committee was not need, but only an informative letter of intent.

### Determination of faecal calprotectin

Faecal calprotectin (FC) was measured using a quantitative immunochromatographic point-of-care test. Calprotectin concentration < 200 μg/g was considered normal reference value.

### Intestinal ultrasonography

IUS was performed by the same expert gastroenterologist (GM, > 3000 IUS per year in the last 20 years), after a 6-h fasting, without any specific bowel preparation. All examinations were performed with the same equipment (Logos Hi-Vision C; Hitachi Medical Systems, Tokyo, Japan) using a broadband convex probe with a frequency of 3.5–5 MHz for a panoramic view of the abdomen and then a high frequency (4–8 MHz) microconvex transducer for detailed bowel evaluation. Despite BWT > 3 mm is the main variable considered for IBD [7], in our Unit IUS is always performed in a systematic way, assessing BWT, bowel wall echopattern, presence of mesenteric lymph nodes and mesenteric hypertrophy.

**Fig. 1:**
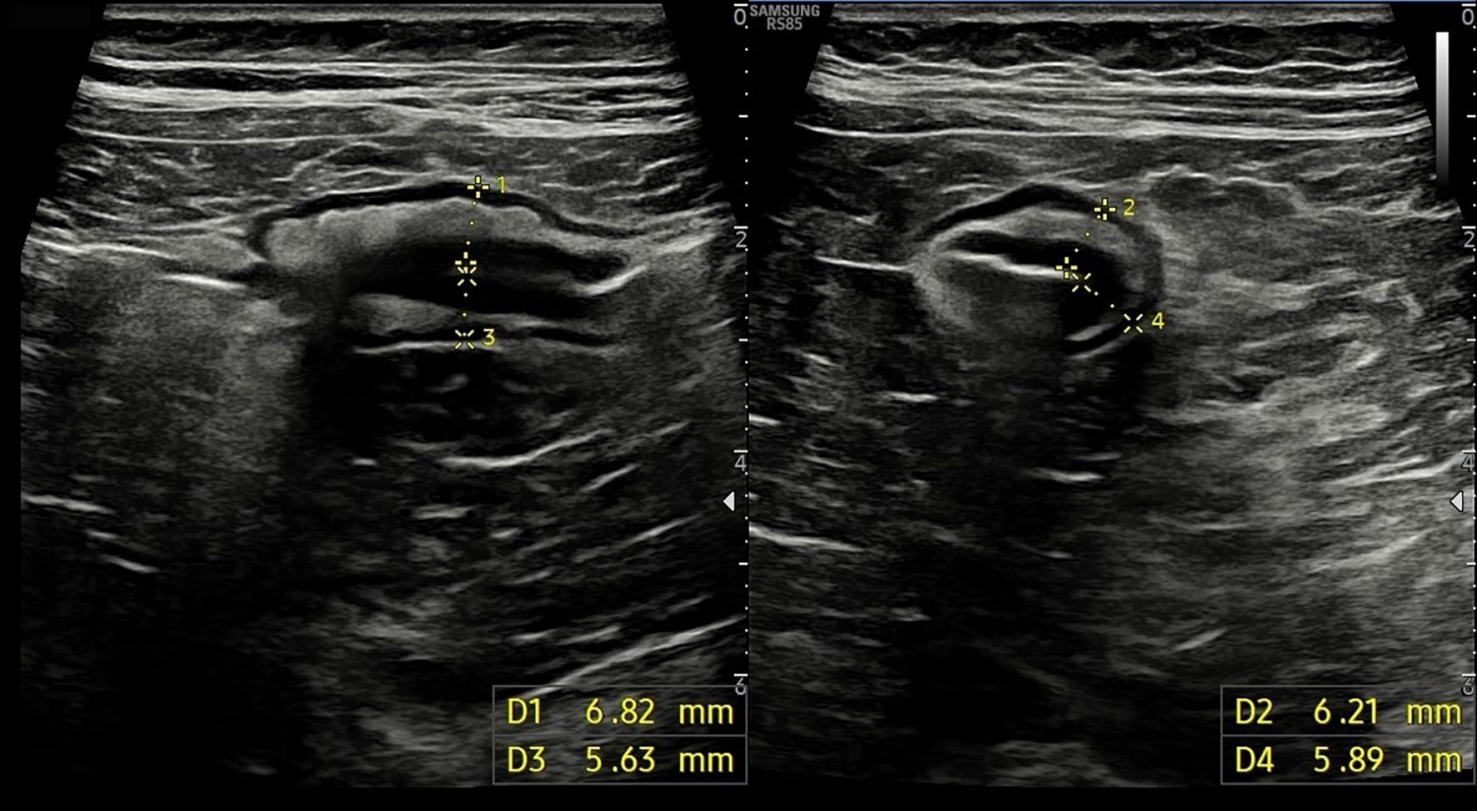
Longitudinal (left panel) and transversal (right panel) scans of small bowel showing a pathological bowel wall thickening (> 3 mm)

**Fig. 2:**
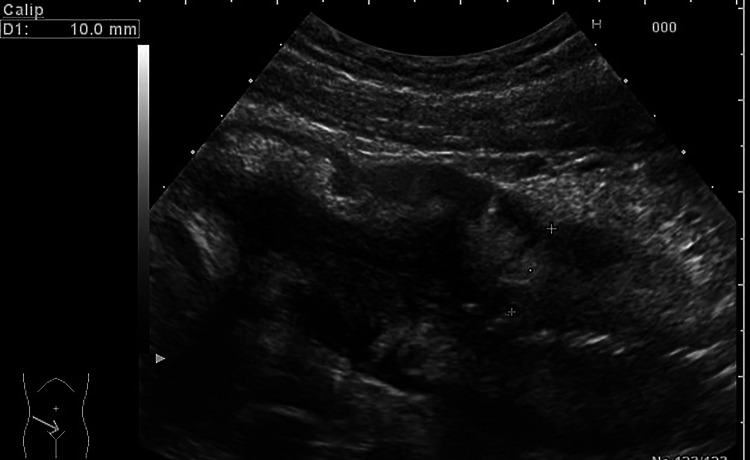
Longitudinal scan of the small bowel showing a pathological wall thickening (10 mm) with preserved stratification

**Fig. 3:**
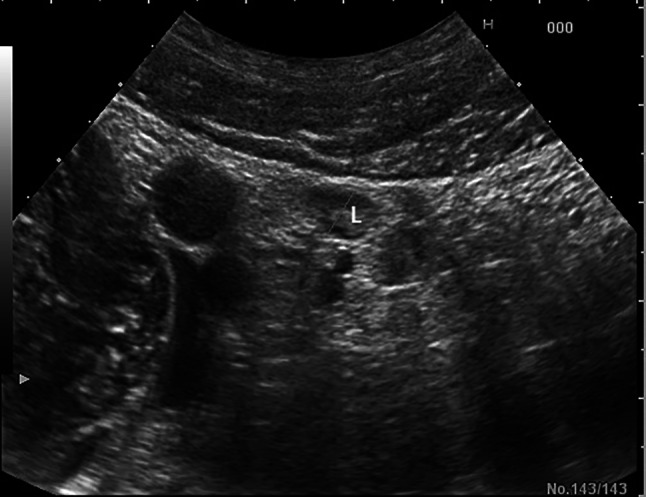
Small reactive regional lymph nodes (L) with lesser diameter of 6–7 mm in the right lower quadrant

**Fig. 4:**
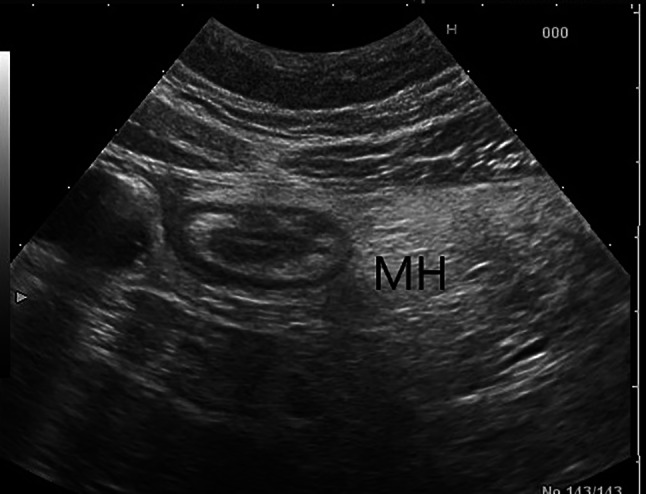
Mesenteric fat hypertrophy (MH) was defined as hyperechoic halo surrounding partly or circumferentially the bowel loops for an extension > 10 mm in a transversal scan

Therefore, BWT was measured perpendicular to the wall from internal interface between the lumen and mucosa to the external interface between serosa and muscularis propria, both in longitudinal and transverse sections, in the most affected intestinal segments, taking the average measurement and considering it pathological if > 3 mm [8, 9](Fig. 1). Bowel wall echopattern was regarded as preserved if normal stratification was visible or disrupted if focal or extensive bowel segments showed disappearance or hypoechoic echopattern [9] (Fig. 2). Reactive regional lymph nodes were reported if lesser diameter was > 7 mm (Fig. 3). Mesenteric fat hypertrophy was defined as hyperechoic halo surrounding partly or circumferentially the bowel loops for an extension > 10 mm in a transversal scan (Fig. 4). Presence of free fluid was also reported if the approximate amount of free peri-intestinal peritoneal fluid was found > 20 ml.

### Statistical analysis

The statistical analysis was performed by a biomedical statistician (AD) using IBM SPSS statistics version 26 (IBM Corp., Armonk, NY). Data are reported as frequencies or means ± standard deviation (SD). Comparisons between groups were made by Mann–Whitney *U* test for continuous variables and the chi-square or Fisher’s exact test for categorical variables. Univariate binary logistic regression was used to evaluate variables predictors of the presence of IBD [10]. The assessment of the independent effect of each of these variables was conducted by multivariate binary logistic regression analysis, using the stepwise backward method. All tests were two tailed. A *P* value < 0.05 was considered statistically significant.

## Results

One hundred thirteen patients were enrolled (mean age 10.8 ± 4.0 years, 65 male). Demographic data are shown in Table 1. Most patients complained abdominal pain (86.7%) and/or diarrhoea (54.0%). At the end of the diagnostic work-up, a diagnosis of IBD was made in 23 patients (20.4%; 8 UC, 12 CD and 3 indeterminate colitis). In the remaining 90 patients a diagnosis different from IBD was made, such as recurrent abdominal pain (25), irritable bowel syndrome (IBS) (23), lactose intolerance (12), functional constipation (8), functional dyspepsia (4), esophagitis (2), acute gastroenteritis (2), gastro esophageal reflux disease (GERD) (2), antibiotic-induced diarrhea (1), gastritis (1), celiac disease (1), pinworm infection (1), C. difficile infection (1), appendicitis (1), cyclic vomiting (1), Helicobacter pylori infection (1), cyclic vomiting + IBS (1), Hp + GERD (1), functional diarrhea (1), cow’s milk protein allergy (1).

**Table 1:** Patients’ characteristics

Characteristic	
Age (years) (mean, SD)	10.8 ± 4.0
Sex (male/female; *n*, %)	65 (58) / 48 (42)
IBD family history (*n* [%])	12 (11)
US indications (*n* [%])	
Abdominal pain	98 (86.7)
Diarrhoea	61 (54.0)
Weight loss > 10%	18 (15.9)
Constipations	17 (15.0)
Vomit	15 (13.3)
Fever	14 (12.4)
Bloating	6 (5.3)
Growth delay	6 (5.3)
Final IBD diagnosis (*n* [%])	
Ulcerative colitis	8 (7.1)
Crohn’s disease	12 (10.6)
Indeterminate colitis	3 (2.6)

*US* ultrasonography, *IBD* inflammatory bowel diseases

As expected, IBD patients showed a greater prevalence of abnormal biochemical tests, especially high levels of FC, compared with patients without IBD (95.7% vs 27.8%, *p* < 0.0001).

As regards IUS, IBD patients showed a significantly higher rate of abnormal findings compared to non-IBD patients, with a degree of variability among different parameters (Table 2). Enlarged mesenteric lymph nodes were the most common IUS finding both in IBD and in non-IBD patients (87% vs 54.4%, *p* 0.004), while increased BWT, altered bowel echopattern and mesenteric hypertrophy (MH) were found far more frequently in IBD patients compared to non-IBD patients (83% vs 13%, *p* < 0.001). Finally, the finding of abdominal free fluid was higher in IBD patients (30% vs 11%, *p* 0.044).

**Table 2:** Demographic, biochemical e sonographic features of IBD patients and non-IBD patients

	IBD (*n* = 23)	Non-IBD (*n* = 90)	*p* value
Age (mean ± sd)	12.3 ± 2.8	10.5 ± 4.2	0.015
Sex M (%)	52.2	58.9	0.6
BII (%)	65.2	15.9	< 0.001
Faecal Calprotectin > unl (%)	95.7	27.8	< 0.001
Altered US-BP (%)	78.3	6.7	< 0.001
Mesenteric hypertrophy (%)	65.2	7.8	< 0.001
Mesenteric lymph nodes (%)	87.0	54.4	0.004
Strictures (%)	13.0	0	0.008
Fistula (%)	8.7	0	0.04
Free fluid (%)	30.4	11.1	0.044
BWT > 3 mm (%)	69.6	3.3	< 0.001
BWT > 3 mm + US-BP + MH (%)	56.5	0	< 0.001
BWT > 3 mm or US-BP or MH (%)	82.6	13.3	< 0.001

*IBD* inflammatory bowel diseases, *M* male, *BII* biochemical inflammatory indexes, *US-BP* ultrasound bowel pattern, *BWL* bowel wall thickness, *MH* mesenteric hypertrophy

Therefore, the main and more frequently found IUS parameters at univariate binary logistic analysis and confirmed at multivariate binary logistic analysis were altered bowel patter (OR 9.8, 95% IC 1.6–59) and mesenteric hypertrophy (OR 5.2, 95% IC 1.1–25.1) with a borderline value for the increased BWT (OR 5.4, 95% IC 0.7–40.1) (Table 3).

**Table 3:** Univariate and Multivariate analysis to predict IBD

Variables	Univariate		Multivariate	
	OR (95% CI)	*p*	OR (95% CI)	*p*
Age	n.s	n.s	–	–
Altered US-BP	50.4 (13.9–183.3)	< 0.001	9.8 (1.6–59.0)	0.013
Mesenteric hypertrophy	22.2 (7.0–70.5)	< 0.001	5.2 (1.1–25.1)	0.04
Mesenteric lymph nodes	5.6 (1.5–20.1)	0.009	–	–
Strictures	n.s	n.s	–	–
Fistula	n.s	n.s	–	–
Free fluid	3.5 (1.2–10.6)	0.026	–	–
BW > 3 mm	66.3 (15.5–283.7)	< 0.001	5.4 (0.7–40.1)	0.09

*IBD* inflammatory bowel diseases, *OR* odds ratio, *US-BP* ultrasound bowel pattern, *BWL* bowel wall thickness

As a consequence, the diagnostic accuracy of IUS in discriminating children with IBD from those without IBD showed that BWT > 3 mm had the greatest positive predictive value (84.2, 95% CI 75.9–90.1), while the absence of altered bowel pattern (normal IUS pattern) shows the greatest negative predictive value (94.4; 95% CI 87.9–97.6). Overall, intestinal ultrasonography bowel pattern (IUS-BP), MH and BWT > 3 mm had a sensitivity value of 78.3%, 65.2% and 69.6%, and a specificity value of 93.3%, 92.2% and 96.7%, respectively. The combination of these three parameters increased the positive predictive value (PPV) up to 100%, whilst the absence of any of these IUS findings showed the greatest negative predictive value (NPV) (95.1; 95% CI 88.9–98.1) (Table 4).

**Table 4:** Diagnostic accuracy of the main IUS findings as single or combined parameters

Variables	Se, % (95% CI)	Sp, % (95% CI)	PPV, % (95% CI)	NPV, % (95% CI)
Altered US-BP	78.3 (69.3–85.2)	93.3 (86.6–96.9)	75.0 (65.8–82.5)	94.4 (87.9–97.6)
Mesenteric hypertrophy	65.2 (55.6–73.8)	92.2 (85.2–96.2)	68.2 (58.7–76.4)	91.2 (84.0–95.5)
BWT > 3 mm	69.6 (60.1–77.7)	96.7 (90.9–99.0)	84.2 (75.9–90.1)	92.6 (85.6–96.4)
BWT > 3 mm + US-BP + MH	56.5 (46.9–65.7)	100 (95.9–100)	100 (95.9–100)	90.0 (82.6–94.6)
BWT > 3 mm or US-BP or MH	82.6 (74.1–88.9)	86.7 (78.7–92.1)	61.3 (51.6–70.2)	95.1 (88.9–98.1)

*IUS* intestinal ultrasonography, *US-BP* ultrasound bowel pattern, *BWL* bowel wall thickness, *MH* mesenteric hypertrophy, *Se* sensitivity, *Sp* specificity, *PPV* positive predictive value, *NPV* negative predictive value

## Discussion

IUS findings in IBD have been well-known for a long time both in adults [8], and children [9, 11, 12]. IUS provides us crucial information about the disease, such as inflammatory activity or presence of strictures, extension of the disease, and presence of absence of complication. Moreover, IUS is ionizing radiations free, well tolerated by patients and easily repeatable [13].

A recent systematic review has demonstrated sensitivity and specificity of 39–93% and 90–100, respectively, for the diagnosis of IBD in children [12].

In our study, we show that the sensitivity and specificity of BWT > 3 mm, altered US-BP and MH were 70% and 97%, 78% and 93%, and 65% and 92%, respectively. Moreover, the best values of sensitivity and specificity were reached by the presence at least of one of these three parameters, which were 83% and 87%, respectively.

Furthermore, sensitivity and specificity of IUS in the detection of CD small bowel lesions were 75% (95% CI 42–94) and 100% (95% CI 66–100), respectively, in an Italian study conducted on 21 children [14].

Among several IUS parameters, the BWT is the most reliable and usually considered for the diagnosis of IBD and CD in particular [7, 8]. However, the role of other IUS parameters or their combination in assessing presence of IBD is still debated.

The usefulness of the combination of non-invasive tests and IUS has been already demonstrated [15]. A recent study combined BWT, mesenteric inflammatory fat and hyperaemia of the bowel wall in a Simple Paediatric Activity Ultrasound Score (SPAUSS) with an area under the ROC curve of 0.82 (95% CI 0.72–0.92) to establish a diagnosis of IBD. In particular, a score > 7 showed the best sensitivity and specificity in the prediction of the activity of the disease. The OR of BWT between 4 and 6 mm and presence of mesenteric inflammatory fat were 3.79 (95% CI 1.28–11.2) and 5.70 (95% CI 1.52–21.32), respectively [16].

The values in our study were slightly higher. However, we used these parameters for the diagnosis of IBD, and not for not for the evaluation of activity in patients with a known diagnosis of CD. In our study, at the multivariate we have found OR for BWT > 3 mm 5.4 (95% CI 0.7–40.1), for altered US-BP 9.8 (95% CI 1.6–59.0) and MH 5.2 (95% CI 1.1–25.1).

The diagnostic weight of a single IUS parameter and of their combination has already been investigated in celiac disease. Fraquelli et al*.* showed that abdominal free fluid and enlarged mesenteric lymph nodes showed a specificity of 96% and 97%, respectively, whereas dilated small bowel loops with increased fluid content and increased peristalsis had a sensitivity of 92% and 83%, respectively [17].

We found that mesenteric lymph nodes were the commonest IUS findings in children complaining abdominal symptoms. However, along with abdominal free fluid likely the less specific, as it has been observed also in a greater proportion of non IBD patients. These findings are in keeping with the data of the literature showing that, although more frequently encountered in inflammatory conditions, enlarged lymph nodes and small amounts of abdominal free fluid are common, unspecific and sometimes clinically insignificant findings in children [18] but may increase anyway the diagnostic confidence and accuracy of IBD when detected along with other findings such as BWT in different clinical context [19].

On the contrary, increased BWT, altered bowel pattern and mesenteric hypertrophy were found far more frequently in IBD patients and could be considered more specific of an inflammatory disorder like IBD, as also confirmed by our multivariate binary logistic analysis. However, their specific weight in IBD diagnosis is still debated. Most studies carried out in paediatric populations considered BWT as the main parameter for assessing IBD and its course after treatment [20] although other authors report that findings such as vascularity and the echopattern more accurate [9].

The main strength of our study is the evidence that the combination of IUS findings may increase the PPV and the NPV of the IUS. In particular, the combination of these three alterations increased the PPV up to 100%, whilst the absence of any of these IUS findings showed the greater NPV. This could be crucial in children where the setting for colonoscopy could be difficult.

Our study includes several limitations. Firstly, it is a retrospective study, and it has a non-univocal reference gold standard as a comparison. Secondly, the assessment of one parameter may be inevitably influenced by the detection of the others. Finally, it has not been included neither colour Doppler or small intestine contrast ultrasonography (SICUS), now considered relevant methods for assessing IBD both in children [10] and in adults [8, 21].

## Conclusion

IUS could be used for the diagnosis of IBD with high sensitivity and specificity in a large retrospective cohort of children. The combination of BWT > 3 mm, US-BP and MH had a positive predictive value of 100% in our cohort. Therefore, the key massage of the study is that sonographic diagnosis of IBD—or better the “suspected diagnosis of IBD” as the true “diagnosis relies of clinical, biochemical, radiological, endoscopic and histological parameters [22]—is better accurate if not based merely on bowel wall thickening only, as for almost all studies reported in the meta-analyses of the literature [7, 23], but on the combination of different sonographic parameters, as we have shown in this study.

We believe that our findings could be of value for clinical practice and research, in particular to plan new studies in adults, in order to investigate the exact role of intestinal ultrasound in patients with abdominal complaints especially those with lower pre-test probability of IBD.

## Data Availability

Not applicable.

## Abbreviations

FC: Faecal calprotectin
BWT: Bowel wall thickening
IUS: Intestinal ultrasonography
BII: Biochemical inflammatory indexes
IBD: Inflammatory bowel diseases
CD: Crohn’s disease
GI: Gastrointestinal
CRP: C-reactive protein

## Declarations

### Data sharing statement

No additional data are available.
